# Speech adapts to differences in dentition within and across populations

**DOI:** 10.1038/s41598-020-80190-8

**Published:** 2021-01-13

**Authors:** Caleb Everett, Sihan Chen

**Affiliations:** 1 Department of Anthropology, University of Miami, Coral Gables, FL USA; 2 Department of Brain and Cognitive Sciences, MIT, Cambridge, MA USA

**Keywords:** Evolution, Anthropology, Coevolution, Cultural evolution, Social evolution

## Abstract

We test the hypothesis that a specific anatomical feature, the dental malocclusion associated with reduced dental wear, causes languages to adapt by relying more heavily on labiodental consonants. In contrast to previous work on this topic, we adopt a usage-based approach that directly examines the relative frequency of such labiodental sounds within phonetically transcribed word lists and texts from thousands of languages. Labiodentals are shown to be very infrequent in the languages of hunter gatherers, who tend to have edge-to-edge bites as opposed to the overbite and overjet observed in populations that consume softer diets and rely heavily on eating utensils. This strong tendency is evident after controlling for Galton’s problem via multiple methods including frequentist and Bayesian linear mixed modeling. Additionally, we discuss data from Amazonian hunter gatherers with edge-to-edge bites. The languages of these populations are shown not to use labiodentals frequently, or to have only recently begun doing so. Finally, we analyze the speech of English speakers with varying bite types, demonstrating how the sounds used by individuals reflect the same phenomenon. The diverse findings converge on the same conclusion: speech adapts to anatomical differences within and across populations.

## Introduction

Many social and physiological factors impact the sounds used in the world’s spoken languages. Disparities in the biomechanical articulatory ease associated with different sound types, for instance, help yield the clear commonality of some sounds and the rarity of others^1^. Other ease-based effects are more subtle, however, and are best detected with large-scale quantitative tests^2^. While linguists agree that ease-based factors influence how languages use their sounds, it is generally believed that all languages are influenced to the same extent by these and other factors. This traditional assumption is sometimes referred to as the uniformitarian hypothesis, as it implies that the various physiological mechanisms that impact sound usage and sound change apply uniformly across human populations both today and in the past^3–5^.

The uniformitarian hypothesis is not agreed upon uniformly by language researchers, however, and has been called into question by a variety of recent studies. These studies suggest, for instance, that cross-population disparities in the shape of the hard palate yield biases for and against the use of clicks and some vowel types, or that extreme ambient aridity in some environments impacts the ease of vocal cord vibration in subtle ways^6–8^. Less recently, Charles Hockett suggested that labiodental consonants are uncommon in hunter gatherer populations because the diet of such populations is associated with heavy wear that yields an edge-to-edge bite amongst adults^9^. In contrast, populations with softer agricultural diets, who also tend to use utensils and rely on more extensive food preparation, have bites that are generally characterized by overbite and overjet. Overbite refers to vertical overlap between the maxillary and mandibular incisors, while overjet refers to horizontal overlap. Hockett suggested that such characteristics facilitate the production of labiodental consonants. Despite initial skepticism towards Hockett’s ideas, recent findings by Blasi et al. (BEA henceforth) support his key assertion that labiodentals were only commonly used once human populations transitioned to agricultural subsistence^10–12^.

**Figure 1:**
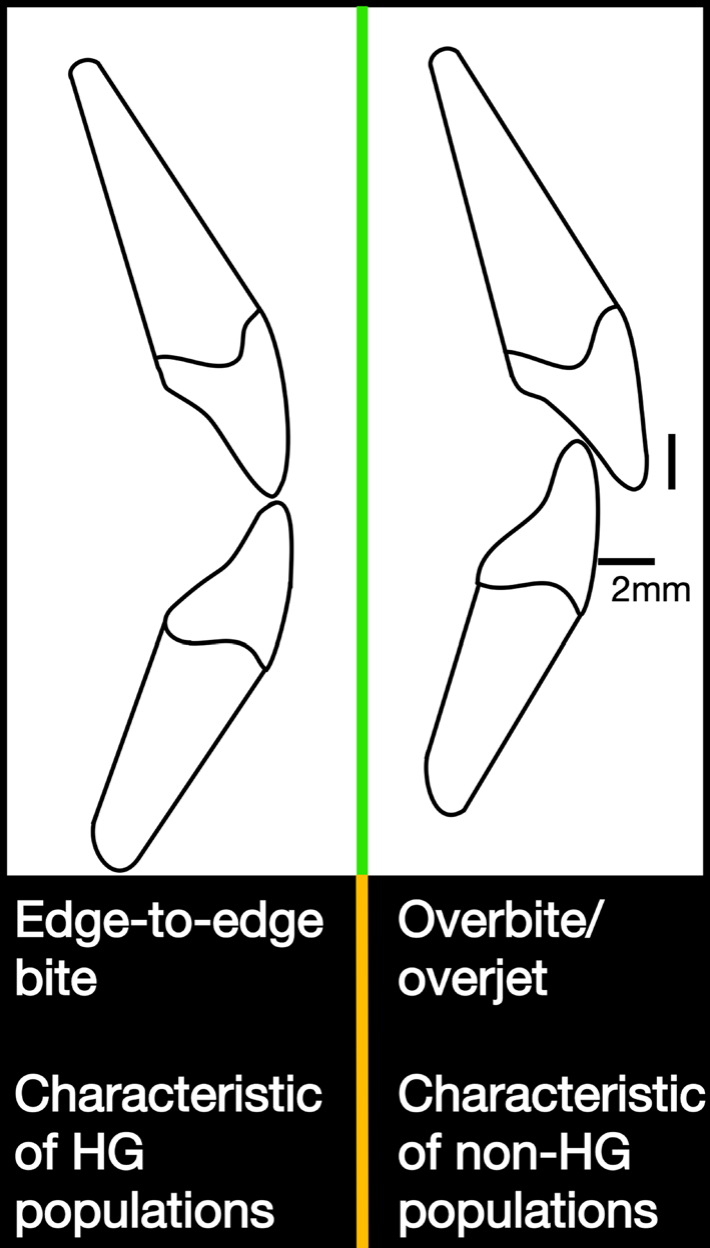
Incisor alignment associated with edge-to-edge bite and overbite/overjet, respectively. Adapted from Brunelle et al.^14^. The vertical black line represents the amount of overbite, the horizontal line represents the amount of overjet. 2 mm represents a common amount of overbite/overjet.

While children generally start life with overbite and overjet, diets with extensive wear reduce these to yield a flatter occlusion. Edge-to-edge bite is due largely to three phenomena during ontogeny, as noted by BEA: lingual tipping, dental eruption, and mesial drift. These factors operate to differing degrees across populations, given the well-known variation in human diet types. As a result, adults in populations that rely primarily on hunting and gathering tend to have edge-to-edge bites, while non-hunter-gathers typically exhibit malocclusion^9,10^. The softer diets of agriculturalists have also been shown to yield slightly smaller mandibles, which may also yield greater rates of overbite and overjet^13^. Overbite and overjet are described visually in Fig. 1. In well-studied populations, clinical measurements of malocclusion support the assessment that softer diets yield overbite and overjet. In one sample of 7000 + individuals, it was observed that only 22% of Americans did not have malalignment of their maxillary and mandibular incisors^14^. The average American overbite was found to be 2.9 mm. Additionally, about 8% of Americans have overbites of 6 mm or greater^14^. In contrast, rates of overbite are much lower in populations of hunter gatherers that have been examined^15,16^. (We refer to such populations as HG populations henceforth).

For individuals with overbite and overjet, the resting position of the bottom lip is closer to the maxillary incisors. Labiodental consonants should require less effort for speakers with such bites, given that labiodentals are produced when the bottom lip moves close to or against the top teeth. This reduction of effort is supported by biomechanical modeling in BEA. However, it is still debatable whether this relative ease of articulation is sufficient to pervasively influence how languages use sounds during speech. Positive evidence for this influence was presented by BEA based on the world’s phoneme inventories. As we note below, there are some limitations to a phoneme-based approach. In the following sections we re-test Hockett’s hypothesis with new methods. Our results suggest that languages use sounds in adaptive ways that pervade speech, further calling into question the uniformitarian hypothesis.

## Results

### The use of labiodental sounds in the world’s languages

According to Hockett and BEA, the languages of HG groups generally do not have labiodental “phonemes”, sounds that are meaningfully contrastive in word sets in a particular language. For instance, the words “bat” and “vat” reveal that the voiced bilabial stop /b/ and the voiced labiodental fricative /v/ are distinct phonemes in English. Phoneme inventories are important but also only offer indirect evidence of the relevant patterns in speech, for a few reasons: First, phonemes can be realized with varied phonetic forms (called “allophones” by linguists), depending on the contexts in which they are produced^4^. Simply because a labiodental phoneme exists in a language does not mean its allophones are always labiodentals. Conversely, a labiodental allophone of a non-labiodental phoneme may not actually be evident in a language’s phoneme inventory. Relatedly, phonemic status is binary but phonemes actually vary dramatically in terms of how well they characterize sound use within a given language. Consider, for instance, the voiced postalveolar fricative, /ʒ/. This sound is phonemic in English but is only contrastive in a few words (e.g. “beige” [be^j^ʒ ] vs. “base” [be^j^s]) and represents about 0.2% of sounds in English speech^17^. In contrast, the alveolar nasal phoneme, /n/, represents about 12.5% of all sounds in English speech, i.e. it is about 63 times as common as /ʒ/^17^. Recent analysis of speech in 32 languages, representing a variety of language families and geographic regions, has found that a sound’s frequency as a phoneme across languages is not always a good indicator of its frequency in speech within a particular language^18^. Some sounds are less frequent in speech than we might expect given their commonality in phoneme inventories. Critically, this was found to be particularly true with respect to the voiceless labiodental fricative /f/^18^. Phonemic status is based on meaning rather than frequency in words, but the proposed reduction of articulatory effort hypothesized by Hockett could only be realized during spoken words. Therefore, some measure of the use of labiodental sounds in words is critical to elucidating this issue. Also, it should be noted that the findings in BEA have been objected to on the grounds that labiodental phonemes are generally less likely to occur in smaller phoneme inventories, and that this confound may help explain their key results^19^. Our approach is not susceptible to this objection.

Given such factors, we examined the rate of occurrence of labiodental sounds in words that are common across the world’s languages, regardless of the labiodentals’ phonemic status in the given languages. (We recognize that the phonemic approach used by Hockett and BEA presents some advantages, as considering phonemic status allows for the examination of the history of labiodentals in particular language families—see the following section.) Our approach also allows us to quantify the pervasiveness of any observed effect within and across languages since it yields continuous variables. We use two methods to examine the rate of occurrence of labiodental sounds in speech, one computational and one based on counting phonetic symbols in texts. The computational approach relies on the Automated Similarity Judgment Program (ASJP), a database of phonetically transcribed word lists for 7000+ documented language varieties, or doculects^20^. For 2729 of these word lists, we were able to obtain three key pieces of information crucial to our analysis, from other databases: 1) Subsistence type associated with the culture represented by the word list, specifically whether or not that culture traditionally relies primarily on hunting and gathering according to the ethnographic record. 2) The language family or isolate represented by the word list. 3) The geographic region of the relevant doculect. (See Methods.) The ASJP includes 40–100 common words for each list, including some very common words for basic pronouns and actions. These words are basically a subset of the Swadesh list developed to test the relatedness of languages^21^. While the extent to which these words reflect wider sound patterns in speech varies across languages, previous work suggests the frequencies of sounds in these word lists are a reasonable proxy for their rates of occurrence more broadly^22,23^. Crucially, larger phonetic corpora are unavailable for most hunter-gatherer languages. As an alternate approach, we examined the narrow phonetic transcriptions of spoken texts provided in the Journal of the International Phonetic Association (JIPA) for 72 languages representing 37 language families in diverse regions. Note that our approach does not aim for phonemic comprehensiveness. It is possible in some cases that a language may have a rare labiodental phoneme and that it may be missing in word lists and/or JIPA transcriptions. Conversely, a language may not have a labiodental phoneme but may have a labiodental allophone in a word list or JIPA transcription. We are not concerned with phonemic status in this study but in ascertaining some value for the frequency of labiodental articulatory gestures in words. The ASJP allows us to do so while testing the bulk of the world’s linguistic lineages.

**Figure 2:**
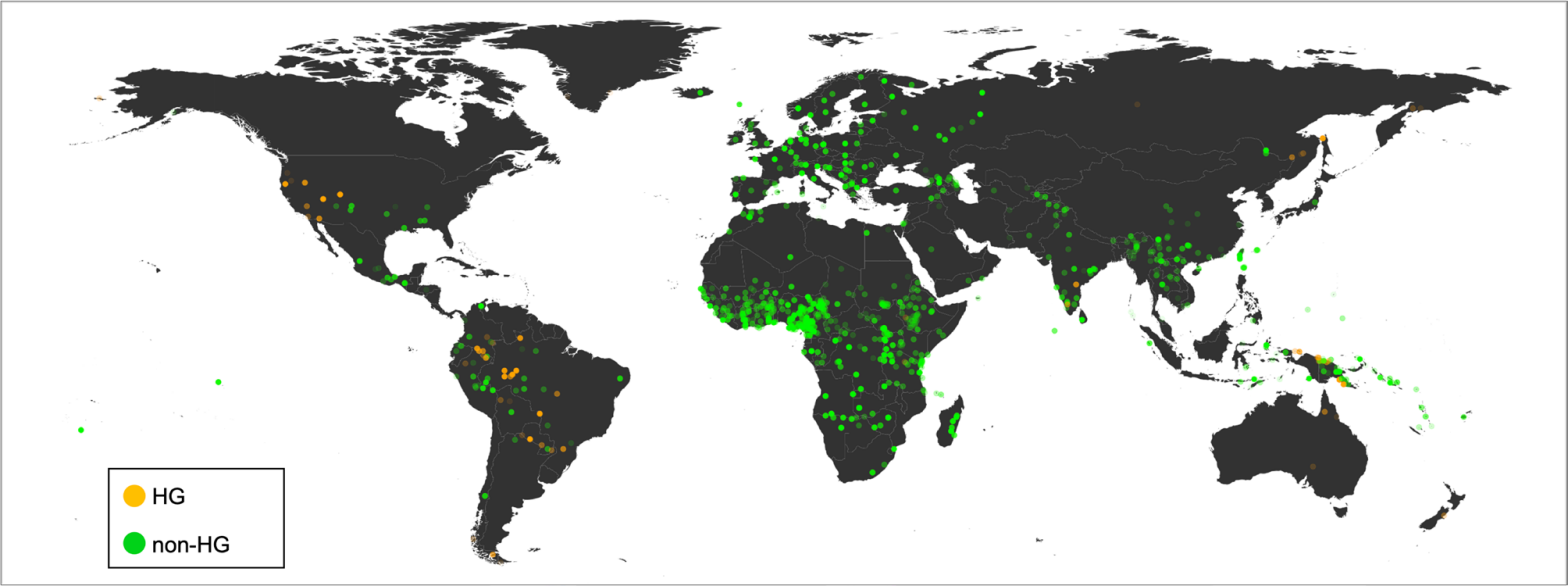
Locations of the 1230 word lists with labiodentals, of the 2729 in the main analysis. Brightness corresponds to LR.

For each word list and text, we tabulated the total number of consonant tokens and the total number of labiodental tokens, and then calculated the ratio of consonants that were labiodental. (Data and code are available in the SI.) This is the “labiodental ratio”(LR henceforth). Nearly all of the labiodental consonants in the JIPA data were labiodental fricatives, and all of the labiodentals in the ASJP database are coded as such. It is worth noting that non-fricative labiodentals are rare in languages, at least judging from phoneme inventories, and that Hockett’s hypothesis as originally formulated actually relates to labiodental fricatives. We also calculated the number of word-initial labiodental consonants for each doculect, dividing this by the total number of consonants. This is the word-initial labiodental ratio (WILR henceforth). The WILR is worth ascertaining because: 1) Word-initial consonants are generally more salient, since sounds towards the beginning of words play a greater role in disambiguating words for listeners^24^. This suggests they are likely transcribed with greater accuracy. 2) Reductive processes are unlikely to occur word-initially^25^. Along with the database that yielded 2729 doculects with subsistence information, we also relied on another smaller database with independent coding of subsistence information for a smaller portion of the dataset^26,27^. Of the 2729 word lists forming the core of the analysis, 1230 have at least one labiodental consonant transcribed. These 1230 doculects are presented on the map in Fig. 2. Of these languages, 1148 belong to languages of non-HG groups. Such figures do not control for confounds such as language family size or the overall higher prevalence of non-HG cultures. Below we control for these and other confounds.

The average LR across all 7221 word lists was about 2% (0.0204), suggesting that Hockett’s hypothesis relates to a pervasive feature of speech worldwide. The LR values for HG and non-HG values are summarized in Table 1. As evident in the table, the doculects of HG groups use labiodentals at about one-third the rate of other groups, on average, according to these data. The doculects of HG groups use word-initial labiodentals about one fourth as much as those of other populations, on average.

**Table 1:** Average LR and WILR values for 2729 ASJP word lists used in the main analysis.

	All word lists		Non-HG languages		HG languages	
	LR	WILR	LR	WILR	LR	WILR
Mean	0.020	0.0106	0.0218	0.0119	0.0079	0.0026
SD	0.029	0.018	0.029	0.018	0.024	0.011

Such results do not control for two major confounds: Language relatedness and language contact^28^. To control for these, we used a mixed methods approach wherein subsistence type was treated as a fixed effect and language family and geographic region were treated as random intercept effects, respectively. Our models utilized logit-transformed values for LR and WILR, given that they are both ratios technically bounded at 0 and 1. We also rely on beta regression for this reason, as described in the SI. We used the AUTOTYP database to categorize doculects into linguistic lineages (families or isolates) and geographic regions. The AUTOTYP database includes 24 geographic regions of known extensive intraregional cross-cultural contact^27^. There are 233 AUTOTYP linguistic lineages represented in the 2729 dialects. Note that AUTOTYP does not include data on labiodentals, those data come from the ASJP. The AUTOTYP data merely served to categorize the ASJP word lists according to taxonomy and geography. We ran the mixed effects tests with the largest categorization of subsistence types, but also separately ran the tests with a smaller alternative categorization of subsistence types that was also based on classifications made in the AUTOTYP database. The latter approach restricted us to 590 doculects (155 lineages, 24 regions). Since we employed two subsistence databases, we used two models to test for the effect of subsistence on LR. Similarly, we employed two models to test for the effect of subsistence on WILR. For each of these models, subsistence was once again treated as a fixed effect, while lineage and geographic region were included as random effects. (See Methods).

The results of the mixed effects tests are summarized in Table 2. The *p* values in the table are based on likelihood ratio tests contrasting each model with a counterpart null model that did not include a fixed effect for subsistence type. Note that for each of the tests HG subsistence has a negative and significant effect on the occurrence of labiodentals. The effect sizes are particularly robust when the AUTOTYP subsistence taxonomy is relied upon. The results are slightly more robust for WILR than LR, regardless of the subsistence taxonomy used.

**Table 2:** Summary of the linear mixed effects models based on logit-transformed LR and WILR values. For the larger subsistence taxonomy, N = 2729 (233 lineages and 24 areas). χ^2^ values based on contrasts between fixed and null models. For AUTOTYP data, N = 590 (155 lineages and 24 areas).

Variable	Subs. tax	Intercept	Coefficient of fixed effect (HG)	χ^2^	*p* value
LR	Large	− 3.260	− 0.144	12.74	0.0004
WILR	Large	− 3.444	− 0.110	14.25	0.0002
LR	AUTOTYP	− 3.245	− 0.215	14.39	0.0002
WILR	AUTOTYP	− 3.448	− 0.132	15.74	< 0.0001

In the SI we summarize the results of two alternative approaches to tackling this issue. One approach is a binomial mixed effects method whereby dialects are categorized in a categorical fashion, as having or not having labiodentals in their word lists. This alternative is useful as it does not require assumptions inherent to linear regressions. The results for the binomial approach are particularly pronounced. The other approach in the SI is a mixed effects method with a Bayesian MCMC estimation, relying on beta regression. We use this Bayesian approach with the same four categories evident in Table 2: LR with the largest subsistence taxonomy, LR with the AUTOTYP taxonomy, WILR with the largest subsistence taxonomy, and WILR with the AUTOTYP taxonomy. The results of this approach also offer support for the hypothesis as the posterior distributions are all skewed in the predicted manner.

**Figure 3:**
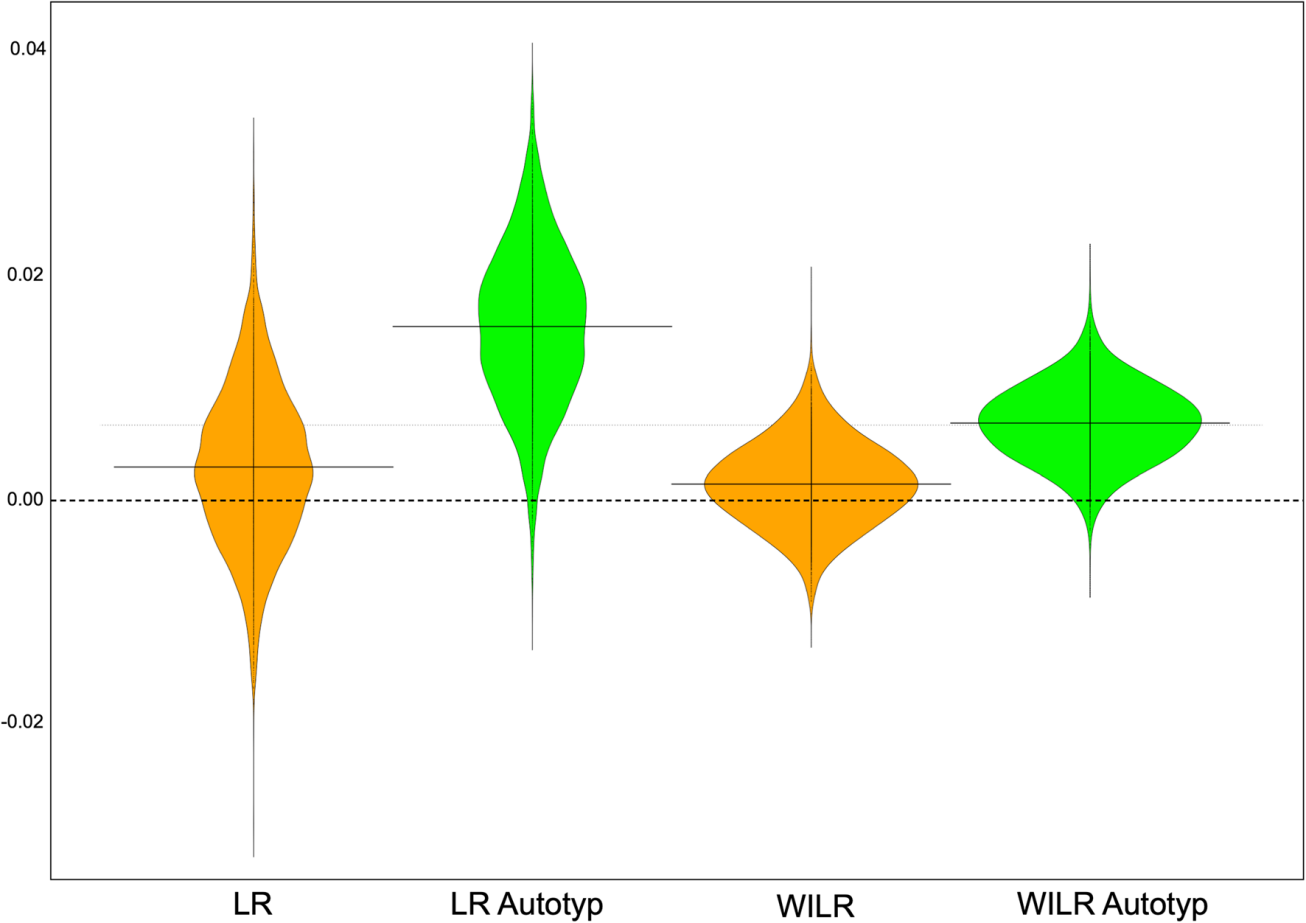
Beanplots for phylogenetically and geographically controlled contrasts between LR and WILR values using two subsistence databases. Each beanplot represents 5000 randomly sampled contrasts, as described in the text. Positive values represent cases in which the values were greater for non-HG groups.

As an alternative to regression-based approaches, we developed a random sampling strategy that allowed us to get a better sense of the pervasiveness of the apparent effect of HG status on labiodental usage. This random sampling approach allowed us to contrast the LR values for HG and non-HG groups while controlling for language lineage and region. It was also used to contrast WILR values across the groups. (See Methods for details.) The sampling method was run 5000 times with each subsistence categorization taxonomy. For each sample contrasting LR values, the mean LR value of HG groups was subtracted from the mean LR value of non-HG groups. For each sample contrasting WILR values, the mean WILR value of HG groups was subtracted from the mean WILR value of non-HG groups. The results across all samples are summarized in Fig. 3. The null hypothesis would be supported by a distributions centered around zero, but this was not observed in any of the four cases. For the larger subsistence classification, the mean LR disparity across all 5000 iterations was positive, 0.0045 (median = 0.0045). For the AUTOTYP subsistence taxonomy, the average LR disparity was again positive, 0.0153 (median = 0.0155). For the larger subsistence classification, the mean WILR disparity across all 5000 iterations was again positive, 0.002 (median = 0.002). For the AUTOTYP subsistence taxonomy, the average WILR disparity was again positive, 0.007 (median = 0.007). The values of the WILR contrasts are lower because word-initial labiodentals are simply a subset of all labiodentals. In the case of WILR contrasts with the larger subsistence taxonomy, the seemingly small average disparity of 0.002 is actually about the same as the average WILR value for all HG doculects (see Table 1), so the difference is not small given the values in question.

Independent analysis of the unrelated JIPA sources once again revealed the pattern: There were 72 languages whose JIPA texts were examined. All of these were non-Indo-European and non-Australian languages. Australian languages are well-known to lack labiodentals fricatives, at least as phonemes, and the associated cultures are uniformly categorized as HG. Indo-European languages typically have labiodentals and the associated cultures uniformly have agriculture. In other words, the choice to exclude Indo-European and Australian languages was motivated and conservative. The 72 languages represent 17 AUTOTYP regions and 37 lineages, giving the sample substantial typological breadth. (See SI for details.) Seven of the languages are spoken by HG groups, and each of these represents a separate lineage. The remaining 65 languages represent 30 lineages. The average LR across all texts in the JIPA data is around 2%, as in the ASJP data. Labiodentals are common in non-HG languages, being found in 18 of 30 lineages. Yet they are found in 0 of 7 lineages spoken by HG groups. This disparity, based on the binary presence/absence of labiodentals in the texts of a linguistic lineage, is significant. (χ^2^ = 8.17, *p* < 0.004) This once again hints that, if Hockett’s hypothesis is correct, the relatively recent development of labiodentals in the world’s languages has had a pervasive effect on speech. None of the seven languages of hunter gatherers in the JIPA data use labiodental consonants of any sort. In contrast, 30 of 65 remaining languages do. The JIPA data are summarized in Table 3.

**Table 3:** Average LR and WILR values for 72 JIPA texts, by language and by language lineage.

Non-HG languages (n = 65)		HG languages (n = 7)	
LR	WILR	LR	WILR
0.020	0.013	0.000	0.000

Non-HG lineages (n = 30)		HG lineages (n = 7)	
LR	WILR	LR	WILR
0.020	0.013	0.000	0.000

### Evidence from Amazonia

One region not considered in depth in previous work on this topic is Amazonia, a critical test case given that it is replete with distinct populations of hunter-gatherers. BEA do present regional analyses of some locations with hunter-gatherers—southern Africa, Greenland and Australia. These analyses suggest that hunter-gatherers with labiodental phonemes have only recently adopted these sounds. Amazonia has yet to be examined carefully, despite a few comments by Hockett. We (CE) have interacted with speakers of numerous language families of HG groups in Amazonia and, based on first-hand impressions and photographs taken by many others, these HG speakers typically have edge-to-edge bites, or at least lack noticeable overbite/overjet. This is consistent with the research on bite types on HG groups, including recent work on Amazonian people^15,16^. That work suggests that, for the populations tested, prevalence of overbite is much more reduced in such populations when contrasted to Americans and others with extensive orthodontic data available^14^. Notably, Amazonian HG languages generally lack labiodentals, judging from their documented phoneme inventories and the ASJP data.

**Figure 4:**
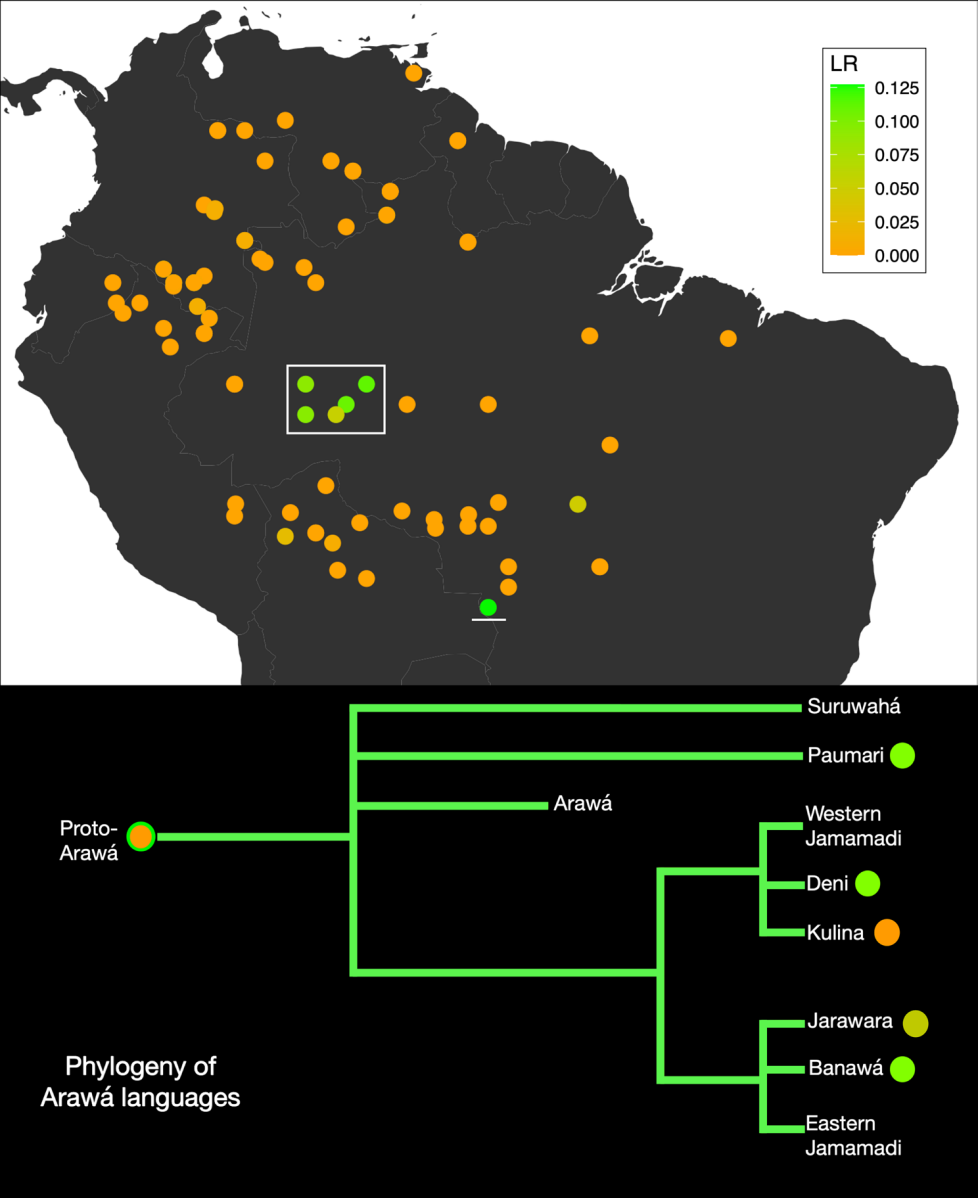
Top panel: map of Amazonian or Amazonia-adjacent doculects of HG groups. Arawá languages highlighted with box. The underlined circle denotes Guato. (See “possible exceptions” spreadsheet in SI.) Bottom panel: Family tree of Arawá languages, based on Dienst (2009), alongside their LRs where available^36^. The shading for proto-Arawá is based on the analysis in Dixon (2004)^33^. (Green border represents uncertainty).

Of all the dialects of hunter gatherers worldwide, only a very small number have LRs and WILRs that exceed the average of agricultural populations. We considered each of these possible exceptions, examining relevant data from historical linguistics when available. (See SI “Possible exceptions” spreadsheet.) Five of the six exceptions to the trend are found in one family in Amazonia, Arawá. We (CE) have previously visited villages of three of the Arawá cultures highlighted in Fig. 4. Our interactions with these cultures include some research on Jarawara number words, and our transcriptions of the language’s numbers include labiodentals^29^. More critically, labiodentals are found in the transcriptions made by Arawá family specialists^30–33^. The prevalence of Arawá labiodentals in the ASJP data and in other sources, including our own work, would appear to suggest that these sounds are ancient in the family. They are not, however, according to a detailed study of sound change in the family^33^. That study suggests the labiodental phoneme in the family, [f], was traditionally a bilabial stop [b] and a bilabial fricative [ɸ]. This change apparently occurred in the late 19th or early twentieth centuries, a point consistent with the transcriptions of geographers and others from that era^34,35^. Notably, this entire family appears to have a shallow time-depth^33^. In short, labiodental phonemes are a recent development in the only Amazonian family in which they are currently well-represented. They surfaced after Arawá speakers had contact with labiodental-using Portuguese speakers. (We cannot determine whether labiodental allophones existed prior to this contact.) This is consistent with Hockett’s hypothesis since he suggested that labiodentals, if present in HG groups, are likely recent innovations owed to language contact and/or population-level changes in diet.

### Evidence from individuals with different bite types

An ease-based bias for/against labiodentals should be evident in the speech of at least some individuals with distinct bites. Studies in linguistics have not considered the role of dental malocclusion on the speech of individuals, but some recent work by dentists and orthodontists has examined the role^37–39^. In one recent study, 115 patients were tested and it was found that an overbite exceeding only 2 mm can lead to multiple speech “errors”^37^. The study uncovered sound production “errors” in about 7% of speakers for a set of sounds including bilabials and labiodentals, and many “errors” for alveolar fricatives. Only 44 of the 115 subjects had overbites less than 2 mm and “normal” sound production. These results suggest that even minor bite-type differences can impact the speech of individuals, perhaps in ways not considered here. Given that many populations do not have average overbites greater than 2 mm, such data support the suggestion that populations face different ease-based pressures during speech.

Since the relevant study offers data on “errors” and does not describe whether, e.g., labiodentals were produced instead of bilabials or vice-versa, we carefully examined the speech of ten famous native English speakers with clearly distinguishable bite types. All ten speakers selected were men, to elimininate any potential sex-based confound. Five of these appeared to have overbite/overjet, though to varying degrees. One had a pronounced overbite. As noted in Section I, about 8% of American speakers have an overbite of 6 mm or greater, so pronounced overbite is not uncommon. None of these five speakers had bites that prevented them from producing bilabial consonants. Five speakers had edge-to-edge bites or modest amounts of underbite. These speakers are less representative of most English speakers, given the comparable rarity of underbite in English-speaking nations. Their bite types did not prevent them from producing labiodentals. All ten speakers’ bites were categorized according to the impressionistic assessments of twelve undergraduate students who were unaware of the hypothesis being tested. The students were presented with close-up pictures of the speakers’ mouths (smiling), and were asked to categorize each mouth as having or not having an overbite. The majority of students’ assessments matched our own, for each mouth (See SI for details).

Phonetic analysis of video recordings of the ten speakers was conducted. While all ten speakers produced both labiodental and bilabial consonants, they exhibited clear differences in their pronunciation of many words, often along the lines predicted by the hypothesis and not predicted by phonological analyses of their English dialects. Speakers in the “overbite” group produced putatively bilabial English sounds as labiodentals in many cases. The speakers with no apparent overbite produced 88 of 4645 total transcribed consonants as labiodentals, while the speakers with overbite produced 365 of 5629 transcribed consonants as such. This disparity was highly significant. (χ^2^ = 127.2, *p* < 0.00001) Video links of these ten individuals are presented in the SI. The speakers’ LRs and WILRs are described in Table 4. (As a point of contrast, the LR for the English ASJP data is 0.059, consistent with the LRs of the speakers with overbite).

**Table 4:** LR and WILR values for ten speakers transcribed, ordered by bite type and LR values.

Speakers with overbite/overjet		Speakers without overbite/overjet	
LR	WILR	LR	WILR
**0.092**	**0.049**	0.062	0.038
0.057	0.024	0.019	0.009
0.052	0.012	0.016	0.008
0.049	0.026	0.010	0.003
0.042	0.021	**0.007**	0.002

Bolded figures are for the two speakers with the greatest perceived difference in bite type.

The relevant speech patterns for these ten speakers are consistent with the pertinent studies in orthodontics that demonstrate speech differences across such bite types. We are not claiming that all English speakers with overbites and edge-to-edge bites should exhibit the patterns in such a clear manner, particularly since varying factors can yield overbites within a given population, including hyperdontia and maxillary prognathism. In fact, one of our ten speakers does not match the larger pattern. Furthermore, the variant bite types evident across these ten speakers are not due to the specific factor first suggested by Hockett, viz. the adoption of agriculture. Nevertheless, speakers like these ten demonstrate clearly that overbite/overjet and edge-to-edge bites can associate with labiodental usage in the predicted manner, even for speakers of the same language. Across approximately ten thousand consonants in speech, these two groups of speakers showed clear evidence of the predicted pattern. Given sufficient individuals with distinct bite types across cultures, it is natural to expect that analogous differences in dentition could yield variable labiodental usage across populations. We believe that they do, as evidenced by the global data we have presented.

## Discussion and conclusion

The various sources of data we have examined point in the same direction. Consonant usage varies in accordance with the bite types of speakers. Labiodentals are relatively uncommon within the languages of hunter-gatherers, who are much more likely to have edge-to-edge bites judging from the data available and given the factors that impact the development of bite types during the lifespan. People with softer diets based largely on rice, wheat, manioc, maize, millet, and other major agricultural staples, are less likely to develop edge-to-edge bites as they age. These groups are also more likely to use utensils, another potential factor contributing to reduction in wear. In fact, it is difficult to dissociate the role that utensil variation, food preparation variation and diet variation play in motivating bite differences across populations. Some research suggests that utensils and extensive food preparation have played a larger role than the introduction of agriculture^10^. All of these factors have become more prevalent in the last few centuries^10^.What is clear is that non-HG groups today have less prevalent edge-to-edge bites than HG groups. This is evidenced by studies of teeth in nations like the US, wherein the average overbite is about 3 mm, and also by scarcer research on the teeth of HG groups^11,15,16^. While genetic factors also impact bite type, it is clear that cross-cultural differences owing to eating behavior also play a role.

Languages are biased towards the usage of sounds that are easier to articulate. Our results suggest specifically that articulatory ease interacts with dental variation to influence the rates at which languages use labiodental sounds. When he first shared his hypothesis, Hockett understood that it would be greeted with skepticism. He observed, already in 1985, that new sources of data would allow for the discovery of correlations between linguistic and nonlinguistic factors, and that some of these were likely spurious. Like Hockett, we agree that there are grounds for skepticism regarding correlations if there is no known mechanism that might yield them. In the case at hand, however, there is an uncontroversial broader mechanism at work, ease of articulation. In addition, in the case of this specific hypothesis a variety of data types converge in a supportive manner, across this study and BEA: (1) Labiodental usage within and across cultures. (2) Word-initial labiodental usage. 3) The speech of individuals with overbites and edge-to-edge bites, from the ten examined carefully here to those in the recent orthodontics literature. (4) Historical data for Amazonia, Africa, Greenland, Australia, and Europe, along with a few other regions given cursory examination by Hockett. (5) The results from biomechanical modeling. (6) The global distribution of labiodental phonemes. (7) The systematic documentation of overbite/overjet in industrialized populations with soft diets. We believe Hockett’s hypothesis offers the most parsimonious explanation of the relevant data. This interpretation implies that languages do indeed face differing external pressures that impact how they evolve in non-uniform ways. It appears that languages use their sounds in ways that adapt to the dental characteristics of their speakers, characteristics that are in turn often due to cultural factors.

## Methods

Statistical analyses were conducted with R^40^. Packages used: stringr, lme4, brms, ggplot, and ggmap^41–45^. All data and code is available via a link in the SI. The code that extracts the LRs and WILRs from the ASJP data, along with those data, are available via the link. The consonants of the ASJP are coded with computationally friendly symbols that represent phonetic units of the International Phonetic Alphabet, as described by the creators of the database^46^. Sequences such as prenasalized segments were treated as two consonants for the purpose of this analysis. The SI contains the words for each ASJP data point, alongside the LRs and WILRs for both the ASJP and JIPA data points, as well as subsistence and language taxonomy info. (See “labiodentals data” spreadsheet.) The SI also includes more detailed data for each JIPA text. For the JIPA analysis, SC read each of the relevant texts and calculated the LRs and WILRs.

The 40 basic words common to the bulk of the ASJP lists are best translated as: eye, ear, nose, tongue, tooth, hand, knee, blood, bone, breast, liver, skin, louse, dog, fish, horn, tree, leaf, person, name, sun, star, water, fire, stone, path, mountain, night, drink, die, see, hear, come, new, full, one, two, I, you, and we.

The JIPA and ASJP values for LRs and WILRs were cross-referenced with the AUTOTYP database via the doculects’ ISO language identification codes and glottocodes. For the larger subsistence categorization, we relied on the same source as in BEA^26^. This categorization is based on a list of languages for which we can confidently claim hunting and gathering as the traditional main form of subsistence. All languages not included in this list are then categorized as belonging to agriculturally reliant groups. The AUTOTYP categorization is based on a separate assessment of each language variety. As BEA note, in this taxonomy cultures are categorized as either “hunting/fishing/gathering/foraging” or “food production”. The assessment is made according to how the population acquired the majority of its calories, at least until recently.

The lme4 tests were also run with an alternate classification of language families and geographic regions, based on the Glottolog database^47^. The results did not differ notably, and remained significant in all cases. We focus here on the AUTOTYP results given its more fine-grained geographic regions.

Each of the mixed effects models took one of the following two forms:

$$\begin{aligned} & \left( {\text{i}} \right)\,{\text{LR}}_{i} =\upbeta _{{{\text{SUBSISTENCE}}}} + \upalpha _{{{\text{FAMILY}}}} + \upalpha _{{{\text{REGION}}}} +\upvarepsilon \\ & \left( {{\text{ii}}} \right)\,{\text{WILR}}_{i} =\upbeta _{{{\text{SUBSISTENCE}}}} + \upalpha _{{{\text{FAMILY}}}} + \upalpha _{{{\text{REGION}}}} +\upvarepsilon \\ \end{aligned}$$

For the random sampling tests, the dialects were separated into two groups–an HG group and a non-HG group. One word list from each of the represented language families in the HG group was randomly selected, and from this sample one list from each of the represented geographic regions was randomly chosen. The average LR was found for this phylogenetically and geographically controlled sample, sample A. Simultaneously, the same approach was used for the non-HG group, yielding a separate phylogenetically and geographically controlled sample, sample B. The average LR for sample A was then subtracted from the average LR for sample B. This random sampling approach was iterated 5000 times for the larger subsistence taxonomy and 5000 times for the AUTOTYP subsistence taxonomy, for both LR and WILR. Positive values for an iteration are consistent with the hypothesis.

SC transcribed several minutes of speech for all ten speakers. Amount of speech transcribed depended on the length of the interview, which was chosen based on the visibility of a speaker’s mouth and overall image quality. Each minute of speech required about 1 h of phonetic analysis, and videos were played at one-quarter speed. These transcriptions were spot-checked by CE. SC’s complete transcriptions are available as pdf files in the SI, alongside video links to the speech segments he transcribed. As evident in the SI, speakers had idiosyncratic productions consistent with their bite type. For instance, the speaker with the most pronounced overbite articulated the bilabial stop, /b/, as a labiodental fricative, [v], in most cases. He also produced the labiodental nasal [ɱ] instead of [m] in some cases. In contrast, one speaker with a modest underbite produced all bilabial sounds as true bilabials, but also produced English labiodental consonants as bilabials in most cases. In some cases speakers produced different variants for the same phoneme in the same word. For instance, speakers in the overbite category consistently produced the first sound in “for” as [f], while speakers in the other category often produced it as [ɸ]. To cite another example, the speaker with the most salient overbite often pronounced the pronoun “we” with a voiced labiodental fricative [v] instead of a bilabial approximant [w]. Note that the pronoun “we” and the preposition “for” are very frequent in English discourse.

## Supplementary Information

Supplementary Information
