# Supplementary material for: Speech adapts to differences in dentition within and across populations
Source: Sci Rep. 2021 Jan 13;11:1066. doi: 10.1038/s41598-020-80190-8 (PMC7806889; doi:10.1038/s41598-020-80190-8)
Supplement: Supplementary file 1 — Supplementary Information [file 41598_2020_80190_MOESM1_ESM.pdf]

SUPPLEMENTAL INFORMATION

"Speech adapts to differences in dentition within and across populations"

Authors:

1. Caleb Everett[a]
caleb@miami.edu

2. Sihan Chen[b]
sihanc@mit.edu

Affiliations:

[a]University of Miami, Department of Anthropology
[b]MIT, Department of Brain and Cognitive Sciences

Data and code can be found at https://osf.io/xt76d/

Contents:

1. Results for mixed effects binomial regression
2. Results for Bayesian mixed effects tests with beta regression
3. Data sheet used in the analysis, with subsistence, lineage, and region information for each doculect ("labiodental data", uploaded separately onto OSF)
4. Data for all 7000+ doculects ("labiodental master", uploaded separately onto OSF)
5. Spreadsheet with comments on potential exceptions, i.e. HG languages with LRs over average of non-HG groups ("possible exceptions", uploaded separately onto OSF)
6. Tabulations of ten speakers' LRs, along with video links (transcriptions uploaded separately onto OSF)
7. Results for undergraduate students' impressions of bite types of ten individuals tested.
8. Code (uploaded separately onto OSF)

1. Results for mixed effects binomial regression. For this approach using the glmer function in R, we categorized all ASJP dialects in a binary fashion. Those that had zero labiodentals in their word lists were coded as 0, while those that had any labiodentals at all were coded as 1. Note that this is not the same as a binary phoneme-based analysis, as there is a mismatch between phonemic status and phonetic presence in word lists (as discussed in the paper). The model and results are summarized as follows:

```
Generalized linear mixed model fit by maximum likelihood (Laplace Approximation) ['glmerMod']
 Family: binomial  ( logit )
Formula: LRbinary ~ Subsistence + (1 | Area) + (1 | Family)
   Data: labiodental_data

     AIC      BIC   logLik deviance df.resid
  2645.3   2668.9  -1318.6   2637.3     2725

Scaled residuals:
    Min      1Q  Median      3Q     Max
-3.4660 -0.4589 -0.1693  0.5031  6.3202

Random effects:
 Groups Name        Variance Std.Dev.
 Family (Intercept) 2.832    1.683
 Area   (Intercept) 1.447    1.203
Number of obs: 2729, groups:  Family, 233; Area, 24

Fixed effects:
                Estimate Std. Error z value Pr(>|z|)
(Intercept)   -0.5291870  0.0005763  -918.2   <2e-16 ***
SubsistenceHG -1.2599668  0.0005764 -2185.9   <2e-16 ***
```

ANOVA contrasting models with and without subsistence

```
MixedtestsofHGlogisticnull: LRbinary ~ (1 | Area) + (1 | Family)
MixedtestsofHGlogistic: LRbinary ~ Subsistence + (1 | Area) + (1 | Family)
                           npar    AIC    BIC  logLik deviance  Chisq Df Pr(>Chisq)
MixedtestsofHGlogisticnull    3 2666.3 2684.0 -1330.1   2660.3
MixedtestsofHGlogistic        4 2645.2 2668.9 -1318.6   2637.2 23.036  1   1.59e-06 ***
```

We did the same thing for WILR:

```
Family: binomial  ( logit )
Formula: WILRbinary ~ Subsistence + (1 | Area) + (1 | Family)
   Data: labiodental_data

     AIC      BIC   logLik deviance df.resid
  2544.5   2568.2  -1268.3   2536.5     2725

Scaled residuals:
```

```
    Min      1Q  Median      3Q     Max 
-3.3549 -0.5037 -0.1851  0.6728  8.4189 

Random effects:
 Groups Name        Variance Std.Dev.
 Family (Intercept) 2.999    1.732   
 Area   (Intercept) 1.252    1.119   
Number of obs: 2729, groups:  Family, 233; Area, 24

Fixed effects:
             Estimate Std. Error z value Pr(>|z|)    
(Intercept)    -1.4973     0.3266  -4.585 4.54e-06 ***
SubsistenceHG  -1.4972     0.3111  -4.813 1.49e-06 ***

MixedtestsofHGlogisticnullWILR: WILRbinary ~ (1 | Area) + (1 | Family)
MixedtestsofHGlogisticWILR: WILRbinary ~ Subsistence + (1 | Area) + (1 | Family)
                               npar    AIC    BIC  logLik deviance  Chisq Df Pr(>Chisq)    
MixedtestsofHGlogisticnullWILR    3 2569.7 2587.5 -1281.9   2563.7                         
MixedtestsofHGlogisticWILR        4 2544.5 2568.2 -1268.3   2536.5 27.208  1  1.827e-07 ***
```

2. Results for Bayesian mixed effects tests with beta regression. For this approach, LR and WILR were treated as the dependent variable, while subsistence was again treated as a fixed effect and AUTOTYP family and region were treated as random effects using beta regression. Four tests were run: i) LR with larger subsistence taxonomy, ii) WILR with the larger subsistence taxonomy, iii) LR with AUTOTYP subsistence taxonomy, and iv) WILR with the AUTOTYP subsistence taxonomy. We used the brms package in R for these tests, following this general syntax: LR+0.00001 ~ Subsistence + (1|Area) + (1|Family), family="beta" (Code available in OSF link.) Note that the approach requires LR values > 0, so 0.00001 was added to the LRs of all doculects.

For each test, four Hamiltonian MCMC chains were run, with two thousand iterations each. The posterior distributions of the intercept effect for HG subsistence is depicted in the following four figures, each of which is followed by the relevant verbose results. In all four cases, well over 99% of the posterior distribution is less than zero, consistent with the hypothesis.

i) Posterior distribution of HG effect on LR, using the larger subsistence taxonomy:

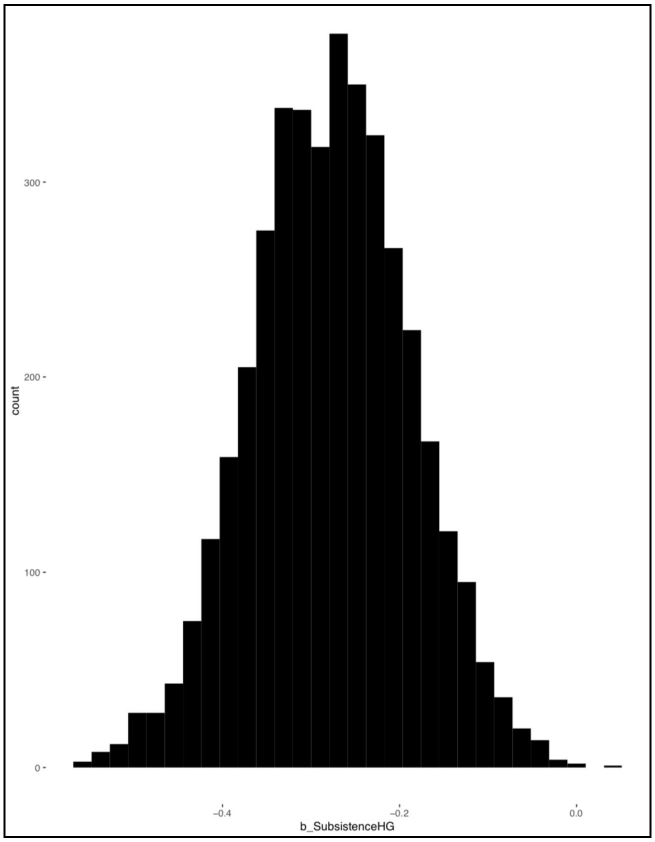

Number of observations: 2729
Samples: 4 chains, each with iter = 2000; warmup = 1000; thin = 1;
total post-warmup samples = 4000

Group-Level Effects:
~Area (Number of levels: 24)

	Estimate	Est.Error	l-95% CI	u-95% CI	Rhat	Bulk_ESS	Tail_ESS
sd(Intercept)	0.43	0.08	0.30	0.62	1.00	1939	2668

~Family (Number of levels: 233)

	Estimate	Est.Error	l-95% CI	u-95% CI	Rhat	Bulk_ESS	Tail_ESS
sd(Intercept)	0.43	0.05	0.34	0.52	1.00	1504	2756

Population-Level Effects:

	Estimate	Est.Error	l-95% CI	u-95% CI	Rhat	Bulk_ESS	Tail_ESS
Intercept	-4.18	0.11	-4.39	-3.96	1.00	1744	2421
SubsistenceHG	-0.28	0.09	-0.46	-0.10	1.00	4764	3456

Family Specific Parameters:

	Estimate	Est.Error	l-95% CI	u-95% CI	Rhat	Bulk_ESS	Tail_ESS
phi	15.00	0.67	13.73	16.34	1.00	8149	2764

ii) Posterior distribution of HG effect on WILR, using the larger subsistence taxonomy:

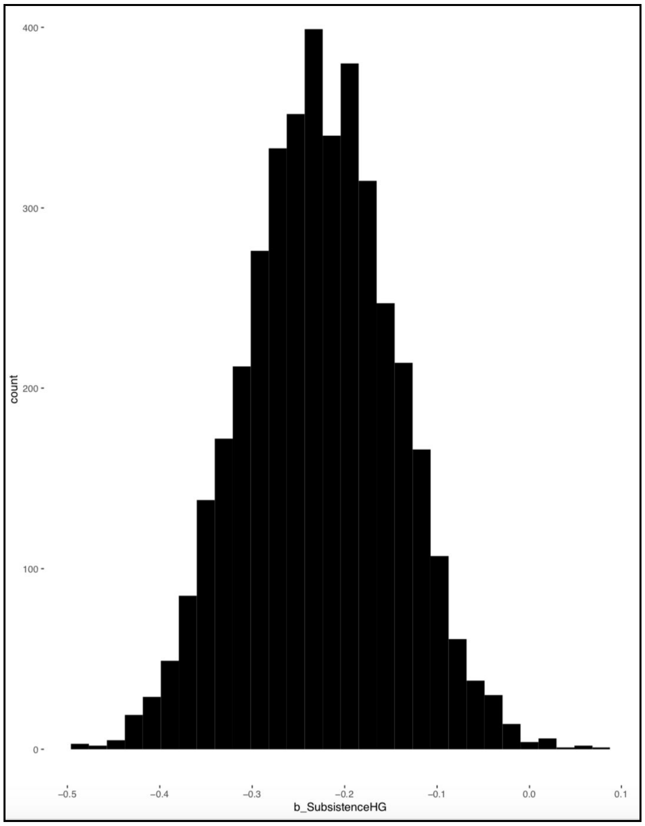

Number of observations: 2729
Samples: 4 chains, each with iter = 2000; warmup = 1000; thin = 1;
total post-warmup samples = 4000

Group-Level Effects:
~Area (Number of levels: 24)

	Estimate	Est.Error	l-95% CI	u-95% CI	Rhat	Bulk_ESS	Tail_ESS
sd(Intercept)	0.33	0.06	0.23	0.47	1.00	1059	2131

~Family (Number of levels: 233)

	Estimate	Est.Error	l-95% CI	u-95% CI	Rhat	Bulk_ESS	Tail_ESS
sd(Intercept)	0.29	0.03	0.23	0.36	1.00	1684	2515

Population-Level Effects:

	Estimate	Est.Error	l-95% CI	u-95% CI	Rhat	Bulk_ESS	Tail_ESS
Intercept	-4.80	0.09	-4.99	-4.62	1.00	959	1720
SubsistenceHG	-0.23	0.08	-0.38	-0.07	1.00	2269	3057

Family Specific Parameters:

	Estimate	Est.Error	l-95% CI	u-95% CI	Rhat	Bulk_ESS	Tail_ESS
phi	22.57	1.04	20.56	24.61	1.00	3411	3056

iii) Posterior distribution of HG effect on LR, using the AUTOTYP subsistence taxonomy:

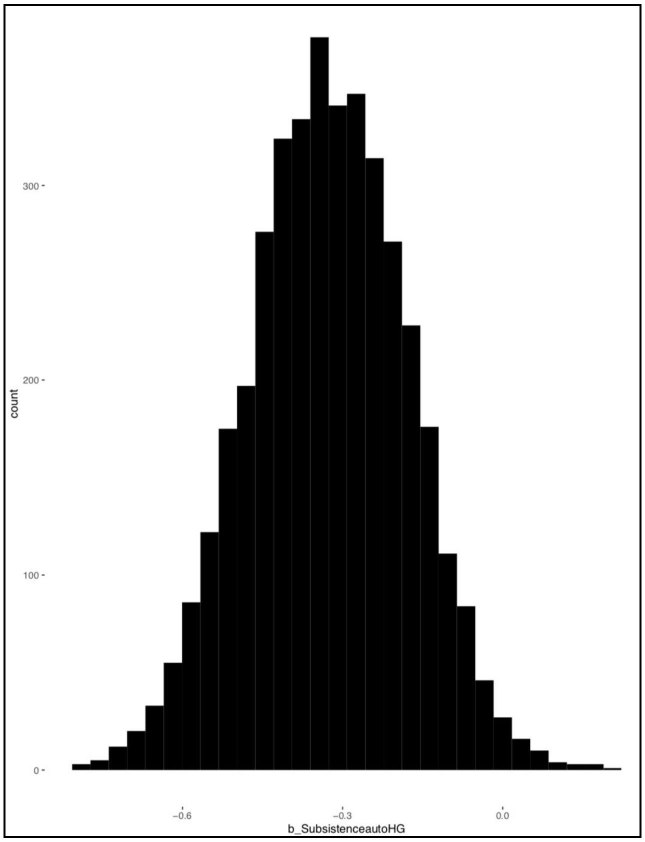

Number of observations: 590
Samples: 4 chains, each with iter = 2000; warmup = 1000; thin = 1;
total post-warmup samples = 4000

Group-Level Effects:
~Area (Number of levels: 24)

	Estimate	Est.Error	l-95% CI	u-95% CI	Rhat	Bulk_ESS	Tail_ESS
sd(Intercept)	0.59	0.18	0.25	0.96	1.01	684	872

~Family (Number of levels: 155)

	Estimate	Est.Error	l-95% CI	u-95% CI	Rhat	Bulk_ESS	Tail_ESS
sd(Intercept)	0.41	0.10	0.18	0.59	1.01	606	970

Population-Level Effects:

	Estimate	Est.Error	l-95% CI	u-95% CI	Rhat	Bulk_ESS	Tail_ESS
Intercept	-4.30	0.17	-4.65	-3.97	1.00	1987	2316
SubsistenceautoHG	-0.33	0.15	-0.61	-0.05	1.00	1959	2523

Family Specific Parameters:

	Estimate	Est.Error	l-95% CI	u-95% CI	Rhat	Bulk_ESS	Tail_ESS
phi	18.90	2.30	14.57	23.62	1.00	1463	2216

iv) Posterior distribution of HG effect on WILR, using the AUTOTYP subsistence taxonomy:

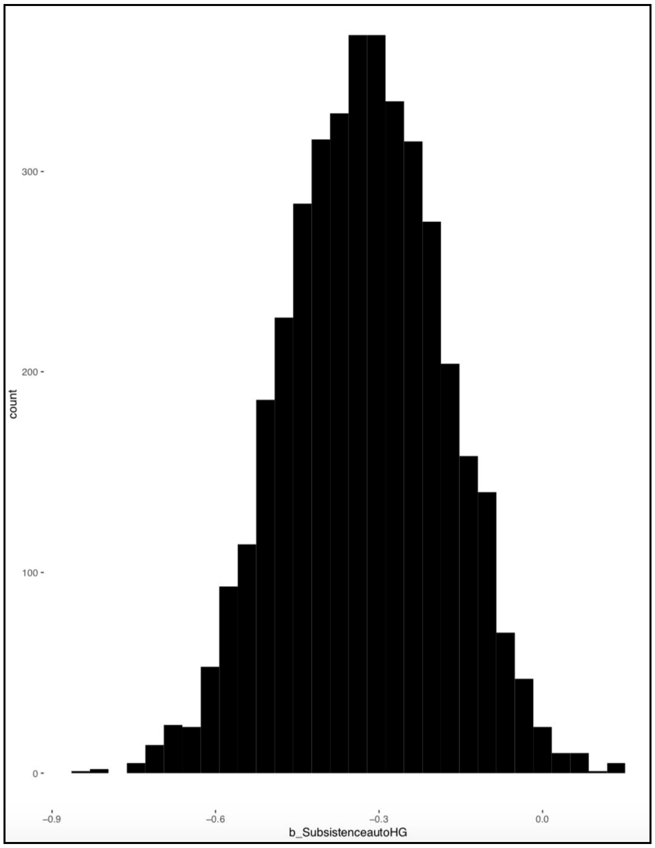

Number of observations: 590
Samples: 4 chains, each with iter = 2000; warmup = 1000; thin = 1;
total post-warmup samples = 4000

Group-Level Effects:
~Area (Number of levels: 24)

	Estimate	Est.Error	l-95% CI	u-95% CI	Rhat	Bulk_ESS	Tail_ESS
sd(Intercept)	0.57	0.18	0.22	0.91	1.00	817	921

~Family (Number of levels: 155)

	Estimate	Est.Error	l-95% CI	u-95% CI	Rhat	Bulk_ESS	Tail_ESS
sd(Intercept)	0.40	0.10	0.18	0.59	1.00	798	885

Population-Level Effects:

	Estimate	Est.Error	l-95% CI	u-95% CI	Rhat	Bulk_ESS	Tail_ESS
Intercept	-4.30	0.18	-4.66	-3.96	1.00	2131	2236
SubsistenceautoHG	-0.33	0.14	-0.61	-0.05	1.00	2939	3212

Family Specific Parameters:

	Estimate	Est.Error	l-95% CI	u-95% CI	Rhat	Bulk_ESS	Tail_ESS
phi	18.82	2.39	14.39	23.67	1.00	1460	2195

3. Data sheet used in the analysis, with subsistence, lineage, and region information for each doculect ("labiodental data")

4. Data for all 7000+ doculects ("labiodental master")

5. Spreadsheet with comments on potential exceptions, HG languages with LRs over zero ("possible exceptions")

6. Ten speakers' LRs and WILRs, along with video links of interviews transcribed. Speakers listed in order from least to greatest LR. Speakers with overbites underlined.

Speaker	LR	WILR	Link for interview transcribed
Michael Phelps	0.007 (16/2223)	0.002 (5/2223)	https://youtu.be/OelU90hH0QA 0:00-9:16
Shannon Sharpe	0.010 (8/800)	0.003 (2/800)	https://www.youtube.com/watch?v=Sm2643Tg1xM 0:00-3:00
Eli Manning	0.016 (8/494)	0.008 (4/494)	https://www.youtube.com/watch?v=WFM_KNotI8c 0:00-2:00
Woody Harrelson	0.019 (6/320)	0.009 (3/320)	https://www.youtube.com/watch?v=XQI8Bp9-Zcc 3:50-8:20
Robin Gibb	0.042 (43/1024)	0.021 (21/1024)	https://youtu.be/Lmuv6r03Uhk 0:20-3:20
Michael Palin	0.049 (47/961)	0.026 (25/961)	https://youtu.be/zX4KsAOBRMU 0:00-3:00
James Carville	0.052 (39/754)	0.012 (9/754)	https://www.youtube.com/watch?v=VQ1yk7QpD5w 0:30-3:30
Tom Cruise	0.057 (47/831)	0.024 (20/831)	https://www.youtube.com/watch?v=AT8OuvIXhRE 0:00-3:00
Conor McGregor	0.062 (50/808)	0.038 (31/808)	https://www.youtube.com/watch?v=q0pszxnsj0E 0:00-2:40
Freddie Mercury	0.092 (189/2059)	0.049 (99/2059)	https://youtu.be/DzooK4wUqMs 1:50-10:40

7. Results for undergraduate students' impressions of bite types of ten individuals tested.

Speaker	Number of students (out of 12) who perceived speaker's mouth to have an overbite
Freddie Mercury	12
Robin Gibb	12
Michael Palin	12
James Carville	12
Tom Cruise	11
Woody Harrelson	5
Michael Phelps	3
Conor McGregor	0
Shannon Sharpe	0
Eli Manning	0

Note: Underlined speakers are those that we considered to have an overbite, based on watching videos of the speakers. The students had only one image and no background on who the individuals were.

8. Code

All code is available via the OSF link on the first page of this SI.
